# Mice Heterozygous for the Sodium Channel Scn8a (Nav1.6) Have Reduced Inflammatory Responses During EAE and Following LPS Challenge

**DOI:** 10.3389/fimmu.2021.533423

**Published:** 2021-03-19

**Authors:** Barakat Alrashdi, Bassel Dawod, Sabine Tacke, Stefanie Kuerten, Patrice D. Côté, Jean S. Marshall

**Affiliations:** ^1^ Department of Biology, Dalhousie University, Halifax, NS, Canada; ^2^ Department of Pathology, Dalhousie University, Halifax, NS, Canada; ^3^ Department of Anatomy and Cell Biology, Institute of Anatomy, Friedrich-Alexander-University Erlangen-Nuremberg (FAU), Erlangen, Germany; ^4^ Department of Ophthalmology and Visual Sciences, Dalhousie University, Halifax, NS, Canada; ^5^ Department of Microbiology and Immunology, Dalhousie University, Halifax, NS, Canada

**Keywords:** multiple sclerosis, inflammation, experimental autoimmune encephalomyelitis, sodium channel, lipopolysaccharide, mast cells

## Abstract

Voltage gated sodium (Nav) channels contribute to axonal damage following demyelination in experimental autoimmune encephalomyelitis (EAE), a rodent model of multiple sclerosis (MS). The Nav1.6 isoform has been implicated as a primary contributor in this process. However, the role of Nav1.6 in immune processes, critical to the pathology of both MS and EAE, has not been extensively studied. EAE was induced with myelin oligodendrocyte (MOG_35-55_) peptide in *Scn8a^dmu/+^* mice, which have reduced Nav1.6 levels. *Scn8a^dmu/+^* mice demonstrated improved motor capacity during the recovery and early chronic phases of EAE relative to wild-type animals. In the optic nerve, myeloid cell infiltration and the effects of EAE on the axonal ultrastructure were also significantly reduced in *Scn8a^dmu/+^* mice. Analysis of innate immune parameters revealed reduced plasma IL-6 levels and decreased percentages of Gr-1^high^/CD11b^+^ and Gr-1^int^/CD11b^+^ myeloid cells in the blood during the chronic phase of EAE in *Scn8a^dmu/+^* mice. Elevated levels of the anti-inflammatory cytokines IL-10, IL-13, and TGF-β1 were also observed in the brains of untreated *Scn8a^dmu/+^* mice. A lipopolysaccharide (LPS) model was used to further evaluate inflammatory responses. *Scn8a^dmu/+^* mice displayed reduced inflammation in response to LPS challenge. To further evaluate if this was an immune cell-intrinsic difference or the result of changes in the immune or hormonal environment, mast cells were derived from the bone marrow of *Scn8a^dmu/+^* mice. These mast cells also produced lower levels of IL-6, in response to LPS, compared with those from wild type mice. Our results demonstrate that in addition to its recognized impact on axonal damage, Nav1.6 impacts multiple aspects of the innate inflammatory response.

## Highlights

- The recruitment of myeloid cells was significantly reduced and levels of IL-10 increased in EAE *Scn8a^dmu/+^*mice.
- *Scn8a^dmu/+^* mice had significantly lower levels of plasma IL-6 during remission and chronic phases, which was associated with less inflammation.
- *Scn8a^dmu/+^* mice demonstrated reduced inflammatory and cytokine responses to LPS challenge.
- Our results suggest a potential general role for Nav1.6 in regulating the inflammatory process.

## Introduction

Multiple sclerosis (MS) is an inflammatory demyelinating disease that affects the central nervous system (CNS) (1) where it causes myelin loss that eventually leads to permanent disability in the majority of patients. Although the root causes of this disease are still unknown, a combination of environmental and genetic factors are thought to be involved (2, 3). T and B cell function, as well as innate immune responses, are believed to play an important role in neuronal damage and loss of the myelin in the CNS (4).

Numerous studies have reported that T cells play a potential role in the immune pathogenesis of MS by crossing through the blood-brain barrier (BBB), which then triggers autoimmune inflammation that destroys myelin (5, 6). Autoreactive T cells produce cytokines that attract inflammatory cells into the CNS, including B cells, natural killer (NK) cells, and monocytes/macrophages. In MS, activated autoreactive myelin-specific CD4^+^ T cells are able to initiate a chronic inflammatory response by migrating into CNS compartments. Autoreactive CD4^+^ T cells cause neurodegeneration leading to a decrease in the neuronal count and grey matter volume (7, 8). Activated macrophages also directly or indirectly cause damage to the CNS by phagocytosing the myelin sheath (9). The innate immune and inflammatory processes required to initiate and sustain disease are driven by a variety of cytokines and chemokines that include a pro-inflammatory cytokine cascade involving TNF and IL-6 (10). These cytokines activate immune effector cells and promote their migration partly through the enhanced expression of adhesion molecules on vascular endothelium (10, 11). A number of regulatory cytokines, such as IL-10 help modulate the inflammatory process (12, 13).

Inflammatory effector cells such as neutrophils contribute to tissue damage in many forms of autoimmune disease including EAE. They play a key role in blood - spinal cord barrier disruption (14) and also have other regulatory roles. The migration and mobilisation of these cells is dependent on inflammatory cytokine signals. Mast cells have also been implicated in EAE (15), although their role is controversial and may depend on the model systems used. However, as key sentinel cells in immunity they provide an excellent model to assess changes in inflammatory cytokine regulation at the cellular level.

Nav channels are transmembrane proteins that can be found in both excitable and non-excitable cells (16). Each Nav channel is composed of one of ten known α-subunit isoforms and one or two regulatory β-subunits (β1, β2 and/or β3) (17). In excitable cells, these channels allow sodium to enter a cell in response to an increase of the voltage across the cell membrane and are essential for the generation of the action potential. In MS, the usually tightly regulated placement and concentration of Nav channels along the axon are profoundly altered following the loss of myelin (18). Our previous work showed that an increase in Nav1.6 in the experimental autoimmune encephalomyelitis (EAE) mouse model of MS allows persistent sodium entry into neuronal cells and is an important factor in eventual neuron death.

While the physiological role of Nav channels in neuronal cells is well characterized (19), these channels are also expressed in many other non-excitable cell types such as glia, immune cells, and some types of cancer cells (20) in which their function is not well defined (21, 22). Nav1.6 is expressed in non-neuronal cells such as astrocytes, microglia, and macrophages as well as in invasive cancer lines (21, 23–25). A significant increase in Nav1.6 expression occurs in activated microglia and macrophages in EAE and MS (25). Also, Nav1.6 has been found to play an important role in initiating microglial migration (22) by promoting the extension of lamellipodia of ATP-activated microglia *via* modulation of intracellular Ca^2+^ levels and a Rac1-ERK1/2 pathway (26).

Our previous work in EAE-induced retina and optic nerve-specific knockouts showed that Nav1.6 contributes to the inflammation in CNS tissue. Here, using heterozygous animals in which EAE was induced by immunization with MOG_35-55_ to induce an immune response against myelin, we extend this investigation by seeking to determine if reduced levels of Nav1.6 regulate inflammation in EAE. We show that in the peripheral blood and optic nerve, the percentage of Gr-1^+^/CD11b^+^cells, and levels of inflammatory cytokines, during EAE, were reduced in the *Scn8a^dmu/+^* mice. Notably, untreated *Scn8a^dmu/+^* mice had levels of the anti-inflammatory cytokines of IL-10, IL-13, and TGF-β1 in the brain that exceeded wild type levels.

*Scn8a^dmu/+^* mice displayed a reduced inflammatory reaction to the well-studied and CNS-independent inflammatory stimulus LPS suggesting a more fundamental dysregulation of inflammatory responses in these animals*. In vitro*, bone marrow-derived mast cells from *Scn8a^dmu/+^* mice, that had been grown in culture independent of the initial host animal for several weeks, also produced lower levels of IL-6 in response to LPS. Our results demonstrate that a reduction in Nav1.6/*Scn8a* expression has a significant inhibitory effect on the inflammatory response through a mechanism associated with dysregulated inflammatory cytokine responses.

## Materials and Methods

### Mice

Two groups of female mice (total *n* = 44), including *Scn8a^dmu/+^* heterozygous (*n* = 22) and *Scn8a^+/+^* wild-type (WT) littermates (*n* = 22), were used in this study. The *Degenerating muscle* (*dmu*) mutation, which is propagated on a C57BL/6 genetic background, consists of a single nucleotide deletion in the sequence coding for the first interdomain loop of Nav1.6, leading to a premature stop codon Homozygous *dmu* mice are not suitable for the induction of EAE due to lethality at approximately three weeks of age (27). Heterozygous *dmu* mice, however, have a similar life-span to wild-type mice and do not exhibit any overt motor dysfunction (28). All animal experiments were approved by the Dalhousie University Committee on Laboratory Animals (protocol no. 17-012 and 19-050). This study was carried out in compliance with the Canadian Council for Animal Care guidelines.

### EAE Induction and Clinical Score

EAE was induced in *Scn8a^dmu/+^* heterozygous (*n* = 10) and *Scn8a^+/+^* mice (*n* = 10) (Hooke laboratories EAE kit, Cat. no. EK-2110). Female mice were injected subcutaneously (s.c.) with 200 μg of MOG_35-55_ suspended in complete Freund’s adjuvant (CFA) containing 4 mg/ml of killed *Mycobacterium tuberculosis* (H37Ra). PTX (Hooke laboratories, Lot no. 1007, 200 ng/mouse) was then injected intraperitoneally (i.p.) on the day of immunization and two days later. Mice were monitored daily for clinical signs of EAE and body weight loss during the course of the disease. Scoring was done by a person blinded to the animal groups, with scores as (1) flaccid tail; (2) hind limb weakness and poor righting ability; (3) inability to right and paralysis in one hind limb; (4) both hind limbs paralyzed with or without forelimb paralysis and incontinence; and (5) moribund. Once mice reached a score of 2 to 3, they were monitored twice daily for dehydration and body weight. These mice were given food supplements (hydration gel and wet chow-slurry), provided with elongated water bottle sipper tubes and/or injected subcutaneously (s.c.) with normal saline as required. Animals exhibiting severe weakness of one or both hindlimbs or paralysis of either or both hind limbs were hand-fed chow-slurry. Animals whose weight fell below 80% of their original body weight or attained a clinical score of 3.5 were euthanized, in accordance with institutional guidelines.

### Blood Collection and Tissues Sampling

**Figure 1:**
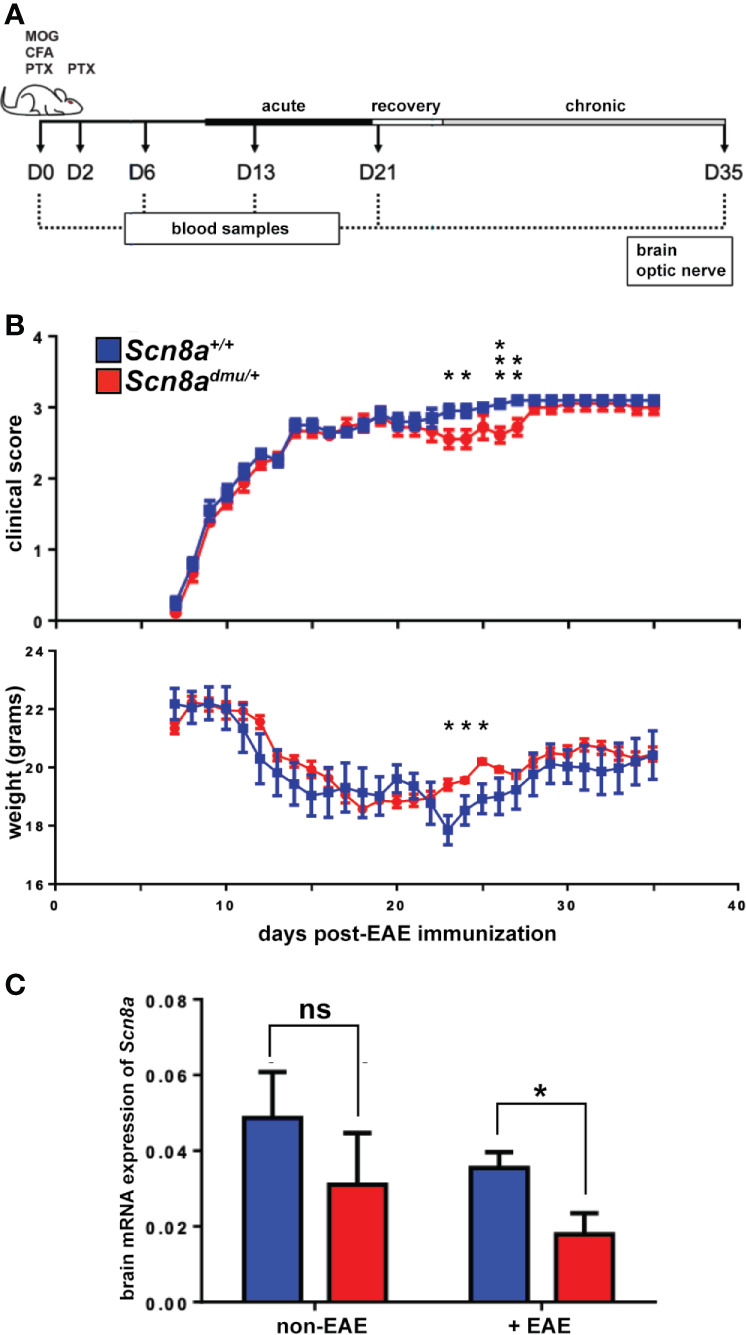
*Scn8a^dmu/+^* heterozygous mice have improved motor function during the EAE recovery and early chronic phase. **(A)** Schematic illustration of the experimental design to evaluate the role of reduced Nav1.6 channels in EAE. Two groups of mice, *Scn8a^dmu/+^* heterozygous and *Scn8a^+/+^* littermates were used in this study. EAE was induced at day 0 in *Scn8a^+/+^* and *Scn8a^dmu/+^* mice by immunization with MOG/CFA followed by PTX and injected at day 2 with PTX only. Mice were sacrificed 35 days post-EAE induction. The clinical score of mice in both *Scn8a^+/+^* and *Scn8a^dmu/+^*mice was recorded daily, starting at day 8 post EAE induction. Blood samples were collected at days 0 (pre-immunization), 6, 13, 21, and 35. On day 35, mice were sacrificed, and optic nerves and brains were collected. **(B)** The clinical score of *Scn8a^+/+^* (*n* = 10) and *Scn8a^dmu/+^* (*n* = 10) mice post -EAE induction revealed a typical progression of MOG-induced EAE but with significantly improved motor function and higher weight in *Scn8a^dmu/+^* in the recovery and chronic stage. **(C)** RNA was extracted from brains of healthy (non-EAE) or chronic stage (35 day) EAE-induced (+EAE) mice. The expression of *Scn8a* in EAE *Scn8a^dmu/+^* was downregulated significantly compared to *Scn8a^+/+^* EAE mice. Data are presented as the mean ± SEM. Mann-Whitney U tests were used to compare clinical scores. Unpaired *t*-tests were used to compare body weights and mRNA expression. *P < 0.05, **P < 0.01, ***P < 0.001, ns, not significant.

Heparinized blood samples were collected from *Scn8a^dmu/+^* and *Scn8a^+/+^* mice (*n* = 14 each) by facial vein puncture on days 0, 6, 13, 21, and 35 post-immunization. The mice were sacrificed at 35 days post -EAE induction (**Figure 1A**). Using flow cytometry, CNS tissues (optic nerve and brain) were studied to define various types of immune cells such as macrophages, myeloid cells, T lymphocytes (CD4^+^ and CD8^+^), and B lymphocytes (CD19^+^). The optic nerves were assessed further by histological staining. ELISA was used to measure the level of several cytokines such as IL-10, IL-13, IL-4, TGF-β1, IL-6, and TNF in the brain and IL-6 in the plasma.

### Staining of Surface Markers and Flow Cytometry

Peripheral blood, spleen, and CNS tissues (optic nerve and brain) from *Scn8a^dmu/+^* and *Scn8a^+/+^* mice (*n* = 14 each) were harvested at different time points. The peripheral blood mononuclear cells were isolated from heparinized blood samples using Ficoll. Cells from spleen and CNS tissues were dissociated by passing through wire mesh followed by addition of an equal volume of 2X enzymatic digestion mix to a final concentration of 10 µg/ml (collagenase D and 100 µg/ml DNase I, Roche Diagnostics, Mannheim, Germany), before incubation in a water bath at 37°C to prepare a single-cell suspension. The suspension was then passed through a 50 micron nylon mesh before analysis to remove debris. The cells were centrifuged at 400 x g for 5 minutes at 4°C and resuspended in 0.5 ml of ammonium chloride lysis buffer (eBioscience, San Diego) on ice for 5 minutes to lyse red blood cells. The cells were centrifuged and resuspended in 1 mL of PBS. Next, the cells were filtered through the mesh for CNS and washed with an additional 1 mL of PBS and counted. Cells were then centrifuged at 400 x g for 10 minutes at 4°C and resuspended in a 1/1000 dilution of fixable viability dye (FVD) eFluor450 (eBioscience, San Diego, CA) in PBS for 30 minutes. The cells were then washed with PBS and stained with specific monoclonal antibodies (mAbs) to define various types of immune cells such as macrophages (F4/80^+^/CD11b^+^), monocytes (Gr-1^int^/CD11b^+^) neutrophils (Gr-1^high^/CD11b^+^/F4/80^-^ or CD45^+^/CD11b^+^/Ly6G^+^), T lymphocytes (CD4^+^, CD8^+^) and B cells (CD19^+^). After incubation, the cells were washed using wash buffer (PBS supplemented with 1% FBS), centrifuged again, and fixed in 1% paraformaldehyde (PFA) in PBS and kept at 4°C. Compensation controls were prepared using compensation beads (eBioscience) mixed with individual dilutions of each antibody used as above in IMF and fixed in 1% PFA. The unstained controls were not incubated with FVD. Stained samples were acquired within 24 hours using a BD FACS Canto™ II cytometer (BD Bioscience, San Jose, CA). All analysis and gating were done using BD FACS Diva software and FlowJo V10.2 (BD Biosciences).

### ELISA

Concentrations of cytokines (IL-6, TNF, IL-10, IL-13, IL-4, and TGF-β1) in plasma samples or homogenized brain were determined using ELISA. We extracted the protein from the brain tissue of EAE mice by homogenizing the samples, using a Qiagen Tissue Ruptor device, in RIPA buffer with protease inhibitor for 1 minute on ice. Samples were centrifuged at 10,000 x g for 10 min, supernatants were collected, and total protein was measured using a Bradford protein assay (Bio-Rad, Mississauga). Levels of cytokines were normalized to total protein concentration. ELISA was then performed according to manufacturer instructions. Briefly, plates were coated with 2.5 µg/ml capture antibody (eBioscience) diluted in borate buffer (pH 8.2). Plates were blocked with blocking buffer (2% BSA in PBS) before samples were added to the plates and incubated overnight at 4°C. Next, biotinylated secondary antibodies (eBioscience) were added to the plate, followed by the addition of streptavidin-horseradish peroxidase. 3,3′,5,5′-Tetramethylbenzidine or substrate solution (TMB) (eBioscience) was added to the plate and incubated for 10–15 minutes before the reaction was stopped using 2N H_2_SO_4_ and the plate measured using an Epoch microplate spectrophotometer (Biotek, Winooski, VT).

### Quantitative Reverse-Transcription Polymerase Chain Reaction (qRT-PCR)

Tissue samples harvested from *Scn8a^dmu/+^* and *Scn8a^+/+^* mice (*n* = 8 each) were immersed in RNAlater (Qiagen, Hilden, Germany). Brain samples were transferred from RNAlater solution (Qiagen, Hilden, Germany) into 700 µL of QIAzol Lysis Reagent (Qiagen). The tissue was homogenized using a Qiagen Tissue Ruptor device. After 5 minutes of incubation at room temperature, 140 µL of chloroform was added to the homogenate, shaken vigorously and centrifuged at 10,000 x g for 15 minutes at 4°C. The upper aqueous layer was extracted, combined with an equal volume of RNase-free 70% ethanol, and RNA was isolated using a Qiagen RNA Mini spin column according to the manufacturer’s protocol. The concentration of RNA in each sample was determined and RNA integrity was determined using a 1% agarose gel to confirm the 2:1 ratio of 28S:18S RNA intensity. RNA samples were reverse transcribed to cDNA using a QuantiTect^®^ Reverse Transcription Kit (Qiagen). The concentration of cDNA extracted from the retina was 100 ng/µL) and from the brain was (500 ng/µL). The cDNA samples were diluted 1:4 and used in qPCR to measure the expression of mRNAs, identifying genes that were implicated in impulse conduction such as Nav1.6 (*Scn8a*) during the clinical course of chronic EAE. Primer sets used are listed in **Table 1**. mRNA expression of the genes of interest was normalized to the geometric mean of the reference *Gapdh* and *Hprt* Cq value expressed as 2-^ΔCq^ where the ΔCq = Cq gene of interest – Cq references genes. Samples were included in each run, without the reverse transcription step, which confirmed the lack of significant genomic DNA contamination.

**Table 1:** qPCR primers.

Gene	Primer sequence or company (catalogue number)
*Hprt*	Bio-Rad (Cat no. 10025636)
*Gapdh*	Bio-Rad (Cat no. 10025637)
*Scn8a*	Forward (5’-3’) GCAAGCTCAAGAAACCACCC Reverse (5’-3’) CCGTAGATGAAAGGCAAACTCT

### Hematoxylin and Eosin Staining of the Optic Nerve

Optic nerves were collected from *Scn8a^dmu/+^* and *Scn8a^+/+^* mice (*n* = 8 each) and fixed with 10% formalin, embedded in paraffin, and sectioned (5 μm) longitudinally. The sections were dehydrated for 2 h at room temperature and fixed for 10 min with 4% paraformaldehyde (PFA) followed by dehydration for 2 min by a series of graded ethanol solutions. The sections were incubated for 5-7 min in hematoxylin and transferred to distilled water. The sections were incubated for 1 min in eosin and dehydrated in a gradient ethanol series before mounting. Images were taken with a Zeiss microscope and software (AxioVision 4.7). Infiltration score was assessed by two investigators blinded to the nature of the samples according to (29) as follows: no infiltration = 0; mild cellular infiltration = 1; moderate infiltration = 2; severe infiltration = 3; massive infiltration = 4. The average number of cell nuclei per mm^2^ was determined for each optic nerve.

### Electron Microscopy

Optic nerve tissues were collected from *Scn8a^+/+,^* and *Scn8a^dmu/+^* mice (*n* = 13 each) sacrificed in the chronic phase, 35 days after induction of EAE. Tissues were then processed according to the process outlined by Alrashdi et al. (30). To demonstrate the axonal loss and myelin pathology, we used a g-ratio that was determined by dividing the axon diameter by the diameter of the myelinated nerve fiber (31, 32). Only axonal g-ratios three standard deviations above (remyelinating) or below (demyelinating) the average of the non-EAE reference group were counted. A conservative margin of ± 3 standard deviations from the mean of normal healthy mice before EAE was used as the cut-off to assign a diagnosis of demyelinating (< 0.55) or remyelinating (> 0.95). The analysis was conducted by an individual that was blinded to the samples.

### Lipopolysaccharide (LPS) Injection

*Scn8a^dmu/+^* and *Scn8a^+/+^* mice (*n* = 12 each were injected intraperitoneally (i.p.) with 100 μL of 50 μg/mL LPS from *Escherichia coli* 055 B5 (Sigma‐Aldrich, Mississauga) or sterile vehicle control (saline). After 16 hours, mice were euthanized and the peritoneal cells were harvested by lavage.

### Peritoneal Cavity Cell Harvesting and Flow Cytometry

Peritoneal cells from *Scn8a^dmu/+^* and *Scn8a^+/+^* mice (*n* = 16 each) were harvested by i.p. injection and recovery of 4 ml PBS, 0.5% bovine serum albumin, 5 mM EDTA. Mouse peritoneal cavity cells (PCC) were counted. Cells were centrifuged at 400 x g for 5 minutes at 4°C and resuspended in a 1/1000 dilution of fixable viability dye (FVD) eFluor 450 (eBiosciences) in PBS for 20 minutes. Then the cells were washed with wash buffer (PBS + 2% FBS + 20 mM NaN_3_), centrifuged and resuspended in antibody cocktails containing anti-CD19-BV510 (clone 6D5, BioLegend), anti-CD11b-PE-eFL610 (clone M1/70, eBiosciences), CD117 (c‐kit)‐PE (clone 2B8, BioLegend), anti-CD4-APC (clone GK1.5, eBiosciences), anti-FcϵRI-APC eFL780 (clone Mar-1, eBioscience), anti-CD8a-BV650 (clone 53-6.7, BD Biosciences), anti-CD3-BV711 (clone 145-2C11, BD Biosciences), anti-Siglec-F-PE-CF594 (clone E50-2440, BD Bioscience), anti-CD11b-PE (clone M1/70, eBiosciences), anti-F4/80-PE-Cy7 (clone BM8, eBioscience), anti-Ly6C-APC-eF780 (clone HK1.4, eBioscience), or anti-Ly6G-BV605 (clone 1A8, BD Biosciences) for 30 minutes. Stained fixed cells were acquired for analysis using a BD LSRFortessa and results were analyzed using FlowJo (Ashland, OR) software. A visual representation of the gating strategy used to identify the cell populations in the mouse peritoneum is provided (**Supplementary Figure 2**).

### Generation of Murine Mast Cells

Bone marrow‐derived mast cells (BMMCs) were generated from the bone marrow of C57BL/6 and *Scn8a^dmu/+^* heterozygous mice as previously described (33). Briefly, mice were sacrificed, the whole femurs and tibias were isolated, and the bone marrow cells were flushed out. The cell suspension was cultured at 0.5–1 × 10^6^ cells/ml in BMMC cell culture media consisting of RPMI 1640 (Life Technologies) supplemented with 10% FCS 10% concentrated WEHI‐3 cell‐conditioned medium 1% penicillin/streptomycin (Life Technologies), 10^−7^ M prostaglandin E2, and 50 μM 2-β-mercaptoethanol (2‐ME). The media was changed twice per week. BMMCs were assessed for purity after four weeks by Alcian blue (pH 0.3) staining of fixed cytocentrifuge preparations and checked for maturity by the expression of c-kit and IgE receptors using flow cytometry. Once the BMMC population reached a purity of >95% (5–8 weeks), they were usedin subsequent experiments.

### Mast Cell Activation

Cells were counted and then resuspended in resting media overnight (BMMC medium with 1.5 ng/mL recombinant IL‐3 instead of WEHI-3 conditioned medium). Activation of BMMC was performed in resting medium with 100 μg/mL of soybean trypsin inhibitor (Sigma‐Aldrich), three doses of LPS (100 μg/mL, 10 μg/mL, and 1 μg/mL), 0.1 μM calcium ionophore (A23187), or in media alone at 10^6^ cells/mL for 24 h at 37°C. Supernatant samples were collected and stored at −20°C until assayed.

### Statistical Analysis

All statistical analyses were performed by unpaired Student’s *t*-tests, Kruskal-Wallis tests, Mann-Whitney U tests, or two-way ANOVA with multiple comparison tests, as indicated and dependent upon appropriate data distribution. The assumption of normality was examined using a Kolmogorov-Smirnov test. Error bars represent the standard error of the mean (SEM). GraphPad Prism software was used for statistical analyses (Ver. 5.0, GraphPad Software, La Jolla, CA).

## Results

To evaluate the impact of reduced expression of *Scn8a*, the gene that encodes the alpha subunit of the Nav1.6 channel, on inflammation in the EAE model, we used *Scn8a^dmu/+^* heterozygous mice and wild type *Scn8a^+/+^* littermates housed in the same cage environment. Mice were immunized with MOG_35-55_, followed by intraperitoneal (i.p.) injection with PTX (**Figure 1A**). The clinical symptoms in all mice subjected to EAE induction appeared 8 days post-immunization. Behavioral observations of *Scn8a^dmu/+^* mice revealed less impact of this treatment on motor capacity during the recovery and early chronic phase of EAE when compared with wild type animals (**Figure 1B**) and during this period, the weight of the *Scn8a^dmu/+^* mice was higher than the wild type. The severity of these symptoms returned to those observed in wild type mice later in the chronic phase (**Figure 1B**). The maximum clinical score observed was 3.5 due to a protocol requirement to euthanize animals at this score. The score of 3.5 was observed in one *Scn8a^dmu/+^* and in two wild type mice.

Real-time qPCR analysis was used to determine how brain *Scn8a* mRNA expression fluctuates in response to EAE in *Scn8a^dmu/+^* and *Scn8a* ^+/+^ mice. In *Scn8a^+/+^* mice we found that in chronic EAE *Scn8a* expression (normalized to two reference genes) was reduced to 71% of their non-EAE level (0.035 ± 0.004 vs 0.049 ± 0.0122), while the *Scn8a* expression in *Scn8a^dmu/+^* in chronic EAE was reduced to 55% of non-EAE levels (0.017 ± 0.005 vs 0.031 ± 0.013). In chronic EAE, *Scn8a* expression in *Scn8a^dmu/+^* was reduced to 49% of *Scn8a* ^+/+^ levels (*P* = 0.028, **Figure 1C**). *Scn8a* mRNA was not detectable by real-time qPCR in leukocytes derived from the spleen or peritoneum (not shown).

**Figure 2:**
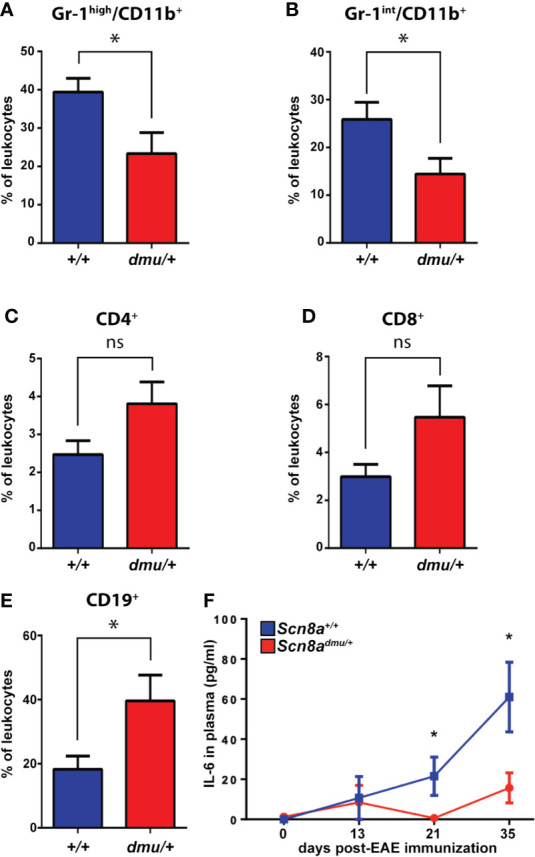
*Scn8a^dmu/+^* heterozygous mice have a lower frequency of Gr-1^high^/CD11b^+^ and Gr-1^int^/CD11b^+^ but a higher frequency of CD19^+^ cells and increase in IL-6 in the blood during the chronic phase of EAE. Immune cell frequency in peripheral blood at 35 days post-EAE from *Scn8a^+/+^* (blue bars, *n* = 8) and *Scn8a^dmu/+^* (red bars, *n* = 8) mice. The frequency of **(A)** Gr-1 ^high^/CD11b^+^, **(B)** Gr-1^int^/CD11b^+^, **(C)** CD4^+^, **(D)** CD8^+^, and **(E)** CD19^+^ cells at days 35 post-EAE induction. **(F)** The level of IL-6 was measured in the plasma of *Scn8a^+/+^* (*n* = 10) and *Scn8a^dmu/+^* (*n* = 8) mice post EAE induction by ELISA. Data are presented as the mean ± SEM. Data are presented as the mean ± SEM. Two‐way ANOVA with Tukey’s *post hoc* test was used to compare the statistical differences among groups *P < 0.05, ns, not significant, unpaired *t*-test.

To characterize the immune cell populations in the blood under normal and pathological conditions, we performed flow cytometry longitudinally throughout EAE. All blood leukocytes were first identified using the general marker CD45 (**Supplementary Figure 1**) before determining the percentage of (Gr-1^high^/CD11b^+^, Gr-1^int^/CD11b^+^), T cells (CD4^+^ or CD8^+^), and B cells (CD19^+^) (**Figure 2**). At 35 days post-induction, representing the chronic phase of EAE, we found a significant reduction (*P* = 0.027) in the percentage of (Gr-1^high^/CD11b^+^) granulocytes in *Scn8a^dmu/+^* mice only reaching 59% of that seen in *Scn8a^+/+^* mice (23.34 ± 5.469%, *n* = 8 vs 39.41 ± 3.549%, *n* = 8; **Figure 2A**). We also found a significant reduction (*P* = 0.035) in the percentage of (Gr-1int/CD11b+) monocytes in *Scn8a^dmu/+^* mice,only reaching 64% of that seen in Scn8a^+/+^ mice (14.40 ± 3.317%, *n* = 8 vs 25.84 ± 3.606%, *n* = 8; **Figure 2B**). In chronic EAE, we found no significant differences in the frequency of CD4^+^ and CD8^+^ T cells in both groups (**Figures 2C, D**). However, in chronic EAE the percentage of CD19^+^ B cells was significantly higher (*P* = 0.034) in *Scn8a^dmu/+^* relative to *Scn8a^+/+^* (39.61 ± 8.078%, *n* = 8 vs 18.25 ± 4.177%, *n* = 8; **Figure 2E**).

The expression of Nav1.6 also influenced the presence of inflammation-related cytokines in the plasma (**Figure 2F**). Following EAE induction, the pro-inflammatory cytokine IL-6 was found to be significantly reduced in *Scn8a^dmu/+^* to 3% of *Scn8a^+/+^* at day 21 (0.63 ± 0.62 pg/mL, *n* = 8 vs 21.51 ± 9.51 pg/mL, *n* = 8, *P* = 0.046) and to 26% of *Scn8a^+/+^* at day 35 (15.70 ± 7.51 pg/mL, *n* = 8 vs 61.01 ± 17.39 pg/mL, *n* = 10, *P* = 0.043). The proinflammatory cytokines TNF and IL-1β and the anti-inflammatory cytokine IL-10 were, however, undetectable in both groups at all time points (not shown).

**Figure 3:**
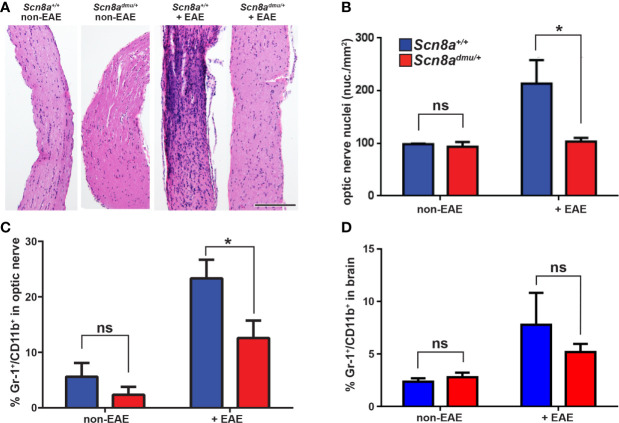
*Scn8a^dmu/+^* heterozygous mice have decreased optic nerve infiltration of Gr-1^+^/CD11b^+^ cells during the chronic phase of EAE. H&E-stained longitudinal optic nerve sections **(A)** and quantification of nuclei per mm^2^ **(B)**. Flow cytometry analysis of infiltration of Gr-1^+^/CD11b^+^ in the optic nerves **(C)** and the brains **(D)** of healthy (non-EAE) and chronic-phase EAE (+EAE, day 35) *Scn8a^+/+^* and *Scn8a^dmu/+^* mice. Scale bar = 500 µm. Data are presented as the mean ± SEM. *P < 0.05, ns, not significant, unpaired *t*-test.

Next, we turned to the CNS and examined how Nav1.6 influences immune cell infiltration in the optic nerve. Longitudinal sections of optic nerves were stained with H&E and cell infiltration was analyzed in *Scn8a* ^+/+^ and *Scn8a^dmu/+^* mice. The total number of nuclei within the optic nerve was significantly higher in *Scn8a* ^+/+^ mice (*P* = 0.041) and numerous cell clusters were seen versus non-EAE *Scn8a* ^+/+^ mice. In contrast, *Scn8a^dmu/+^* optic nerves were relatively devoid of clusters, and total nuclei counts were equivalent to non-EAE *Scn8a^dmu/+^* mice (**Figures 3A, B**). To further analyze the infiltrating cells in the optic nerve and the brain, flow cytometry was performed. The percentage of (Gr-1^+^/CD11b^+^) granulocytes was significantly less (*P* = 0.0100) in the *Scn8a^dmu/+^* mice reaching 54% of that observed in *Scn8a^+/+^* mice (12.58 ± 3.840%, *n* = 8 vs 23.33 ± 3.840%, *n* = 10) at the chronic stage of EAE (**Figure 3C**). The percentage of Gr-1^+^/CD11b^+^ granulocytes in the brain was also found to be substantially reduced in the *Scn8a^dmu/+^* vs *Scn8a* ^+/+^ mice but without reaching significance (**Figure 3D**). The CD8^+^, CD4^+^, and B cell populations in the optic nerve and brain did not change significantly between both groups of mice (data not shown). Using the methods described by Olivera et al. (34) to distinguish resting microglia from activated microglia and macrophages, using expression levels of CD45 to distinguish these populations, we observed no significant differences in these populations. For example, at day 35 post EAE induction resting microglia made up 42.4 ± 3.6% of CD45+ cells in wild type mice and 45.7 ± 4.3% in mice with reduced Nav1.6 expression. Under these conditions, the percentage of CD45+ cells identified as activated microglia/macrophages were 19.6 ± 2.9% in wild type animals and 18.8 ± 2.2% in those with reduced Nav1.6 expression (*n* = 5/group).

**Figure 4:**
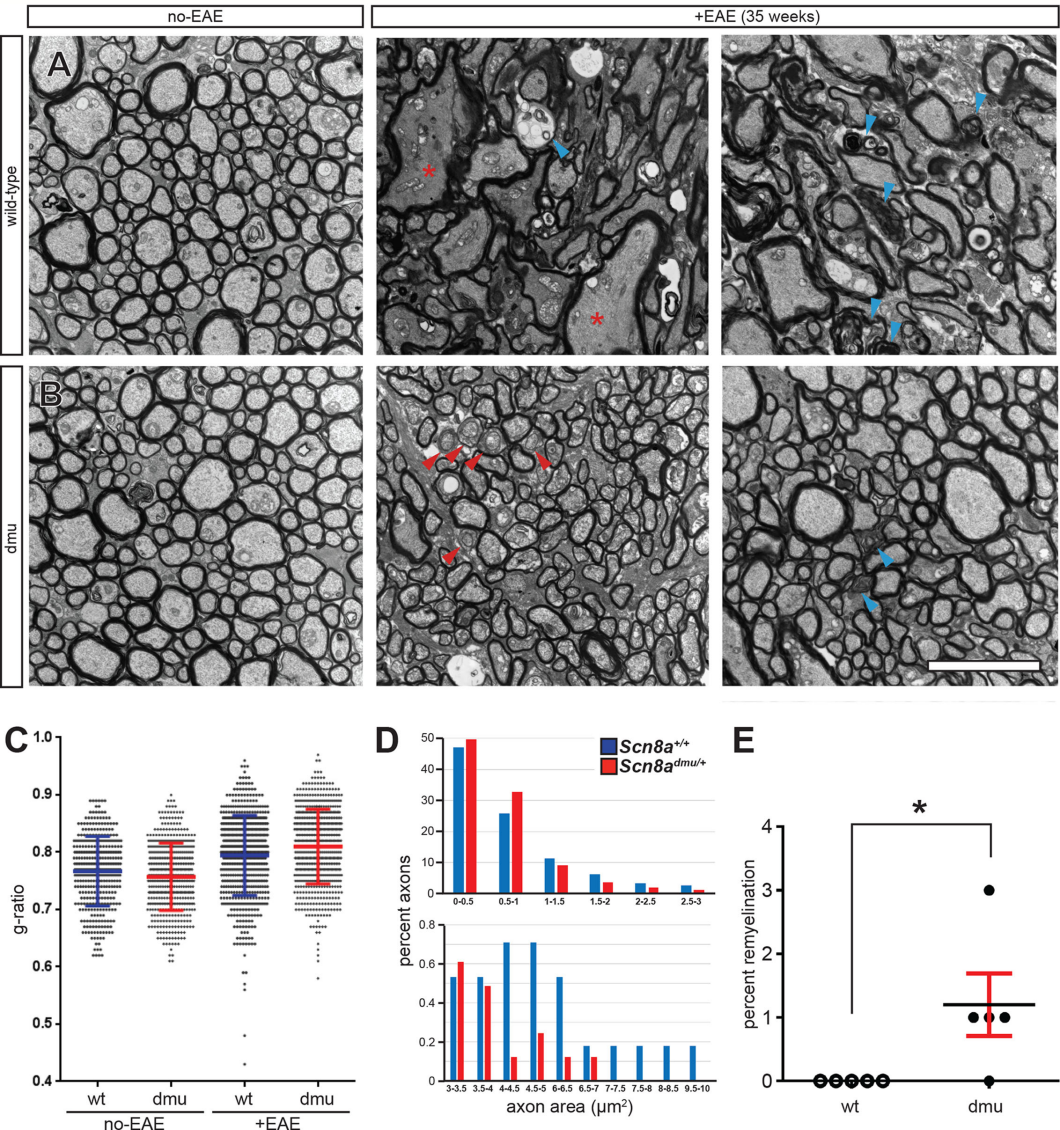
Improved axonal pathology in optic nerves of *Scn8a^dmu/+^* heterozygous mice during the chronic phase of EAE. Representative EM micrographs of healthy (non-EAE) and 35 day EAE (+EAE) optic nerves from **(A)** *Scn8a^+/+^* and **(B)** *Scn8a^dmu^* ^/+^ mice. Red asterisks indicate enlarged fibers, blue arrowheads show axolytic fibers and red arrowheads show remyelinating fibers. **(C)** Summary data for g-ratios from pooled axonal measurements of healthy and EAE optic nerves from *Scn8a^+/+^* and *Scn8a^dmu^* ^/+^ mice (error bars are ± SD). **(D)** Distribution of the axonal area in 35 day post-EAE (error bars are ± SEM). **(E)** Frequency of remyelination. Scale bar, 5 µm. *P < 0.05, unpaired *t*-test.

To assess the effects of Nav1.6 in myelin sheath pathology, we performed optic nerve ultra-structure analysis (**Figure 4**). We found that nerves from healthy *Scn8a^+/+^* and *Scn8a^dmu/+^* (non-EAE) controls had a similar appearance with axons generally appearing rounded throughout the optic nerve (**Figures 4A, B**). Interestingly, the g-ratio (25, see *Materials and Methods*) of healthy *Scn8a^dmu/+^* was found to be significantly lower than in healthy *Scn8a^+/+^* optic nerves (*P* = 0.0196; 0.7572 ± 0.058884; *n* = 483 vs 0.7668 ± 0.060645; *n* = 375; **Figure 4C**). However, in *Scn8a^+/+^* mice at 35 days post-EAE induction, the axons generally appeared deformed. Several pathological features could be identified in EAE mice, including demyelinated and axolytic fibers, though the frequency of these features did not differ significantly between *Scn8a^+/+^* and *Scn8a^dmu/+^* optic nerves. However, a representation of the distribution of the axonal area (measured inside the myelin sheath) revealed that more axons in *Scn8a^+/+^* mice were between 4-10 µm² compared to *Scn8a^dmu/+^* mice and only *Scn8a^+/+^* had very enlarged axons between the sizes of 7 and 10 µm² (**Figure 4D**). The g-ratio post-EAE was significantly higher in *Scn8a^dmu/+^* vs *Scn8a^+/+^* mice (*P* < 0.0001; 0.8097 ± 0.002624; *n* = 618 vs 0.7944 ± 0.002803; *n* = 622; **Figure 4D**). No demyelinating axons were observed in either group (not shown). However, in four out of five *Scn8a^dmu/+^* mice a low yet significant number of axon with g-ratios that are consistent with remyelination (> 0.95) were observed while none were seen in *Scn8a^+/+^* (*P* = 0.0400; 1.200 ± 0.4899%, *n* = 5 vs 0.0 ± 0.0%; *n* = 5) in *Scn8a^+/+^*mice (**Figure 4E**).

**Figure 5:**
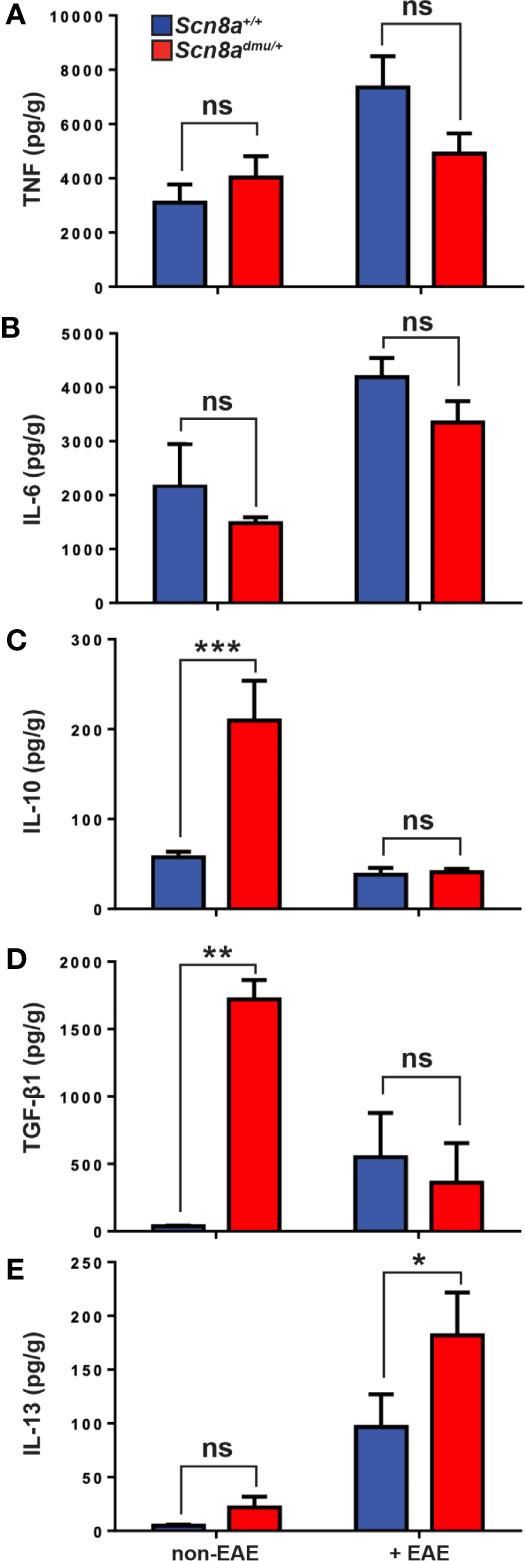
Healthy (non-EAE) *Scn8a^dmu/+^* heterozygous mice express higher levels of regulatory cytokines while IL-13 is increased during the chronic phase of EAE. The levels of **(A)** TNF and **(B)** IL-6 were measured in the brains of *Scn8a^+/+^* and *Scn8a^dmu/+^* mice before (*Scn8a^+/+^*, *n* = 4 vs *Scn8a^dmu/+^*, *n* = 4) and after EAE (day 35; *Scn8a^+/+^*, *n* = 7 vs *Scn8a^dmu/+^*, *n* = 8). The levels of **(C)** IL-10, **(D)** TGF-β1, and **(E)** IL-13 were measured in the brains of *Scn8a^+/+^* and *Scn8a^dmu/+^* mice before EAE (*Scn8a^+/+^*, *n* = 4-7 vs *Scn8a^dmu/+^*, *n* = 3-7) and after EAE (day 35; *Scn8a^+/+^*, *n* = 7 vs *Scn8a^dmu/+^*, *n* = 7). Data are presented as the mean ± SEM of cytokine level per gram of brain tissue. *P < 0.05, **P < 0.01, ***P < 0.001, ns, not significant, unpaired *t*-test.

In the brain, we analyzed pro-inflammatory and anti-inflammatory cytokine levels by ELISA in healthy control and chronic phase-EAE mice. In chronic EAE, the pro-inflammatory cytokine TNF (**Figure 5A**) and (**Figure 5B**) IL-6 responses were both modestly reduced in *Scn8a^dmu/+^* relative to *Scn8a^+/+^* mice but neither change reached significance. The anti-inflammatory cytokines IL-10, TGF-β1, and IL-13, were also examined in the brain by ELISA in non-EAE and in chronic-phase EAE mice. Surprisingly, the levels of IL-10 in non-EAE *Scn8a^dmu/+^* mice were found to be 3.7 fold higher than control non-EAE *Scn8a^+/+^* values (209.6 ± 44.33 pg/mL, *n* = 7 vs 57.28 ± 6.285 pg/mL; *n* = 7, *P* = 0.0002; **Figure 5C**). Similarly, levels of TGF-β1 in non-EAE *Scn8a^dmu/+^* mice were 45-fold higher than control levels (1722 ± 143.3 pg/mL; *n* = 3 vs 37.79 ± 4.597 pg/mL; *n* = 4; *P* = 0.0057; **Figure 5D**). In both *Scn8a^dmu/+^* and *Scn8a^+/+^* mice, the level of IL-10 was lower in chronic-phase EAE while the level of TGF-β1was found to be significantly lower only in *Scn8a^dmu/+^*. As opposed to IL-10 and TGF-β1, we did not find a significant difference for IL-13 in untreated animals. However, in chronic EAE, IL-13 was significantly higher in *Scn8a^dmu/+^* mice than in *Scn8a^+/+^* mice (181.9 ± 39.93 pg/mL, *n* = 6 vs 101.2 ± 33.15 pg/mL, *n* = 6, *P* = 0.048; **Figure 5E**).

**Figure 6:**
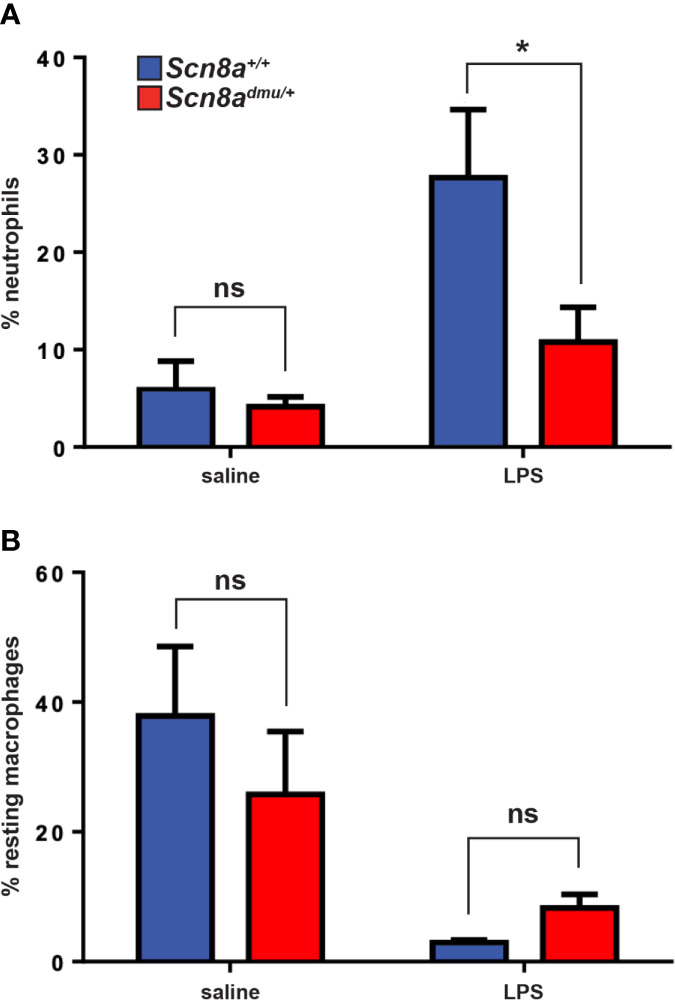
*Scn8a^dmu/+^* heterozygous mice display decreased infiltration of neutrophils in the peritoneum post-LPS stimulation. The frequency of neutrophils **(A)** and resting macrophages **(B)** were analyzed by flow cytometry in the peritoneal lavage of WT and dmu mice before (*Scn8a^+/+^ n* = 4 vs *Scn8a^dmu/+^ n* = 4) and 16 hours post-LPS stimulation (*Scn8a^+/+^ n* = 8 vs *Scn8a^dmu/+^ n* = 8). Data are presented as the mean ± SEM. *P < 0.05, ns, not significant, unpaired *t*-test.

Having demonstrated that mice with reduced levels of Nav1.6 display a reduced inflammatory reaction in the EAE model of CNS inflammation, we aimed to define whether Nav1.6 might be generally involved in the regulation of neutrophils and macrophages in response to inflammatory stimuli, such as LPS insult. Neutrophils are a major immune cell type recruited during acute inflammation. Analysis of peritoneal cavity cells (PCC) following an intraperitoneal injection of LPS (5 μg/animal) resulted in a significant increase in the number of neutrophils after 16 hours in *Scn8a^+/+^*mice. However, this neutrophil response was significantly lower in *Scn8a^dmu^* ^/+^ than in *Scn8a^+/+^* mice at 16 h post-LPS (10.77% ± 3.566, *n* = 8 vs 27.65% ± 6.989, *n* = 8, *P* = 0.0199, **Figure 6A**). The resting monocytes were reduced in *Scn8a^+/+^*mice at 16 h post-LPS (*P* = 0.0054), whereas a more modest reduction was observed in the *Scn8a^dmu^* ^/+^ mice post-LPS without reaching statistical significance (**Figure 6B**).

**Figure 7:**
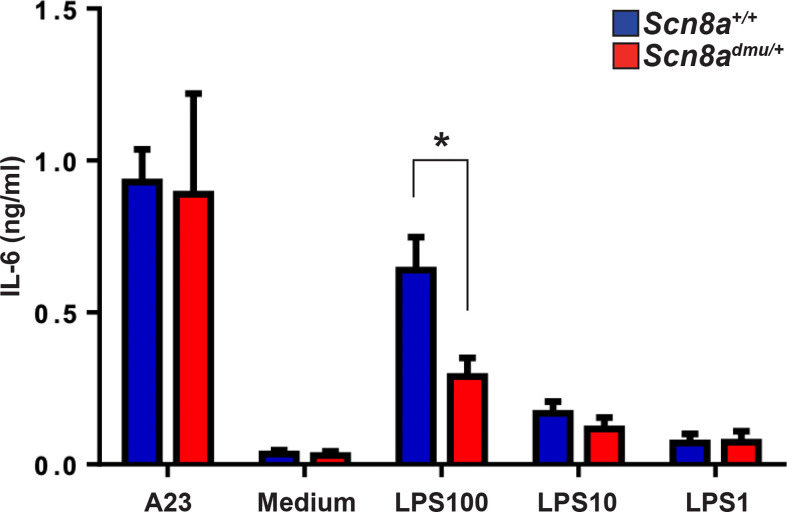
Mast cells from *Scn8a^dmu/+^* heterozygous mice produce lower levels of IL-6 in response to LPS stimulation *in vitro*. Bone marrow-derived mast cells (BMMC) were cultured and stimulated with 100, 10, 1, and 0 µg/ml of LPS or 0.5 μM calcium ionophore (A23187) in the medium for 24 hours. The concentration of IL-6 secreted by *Scn8a^+/+^* (*n* = 5) and *Scn8a^dmu/+^* (*n* = 5) BMMC was measured by ELISA. Data are presented as the mean ± SEM. *P < 0.05, Kruskal-Wallis test.

Reduced inflammatory responses in the presence of low Nav1.6 expression might result either from changes in acquired immune responses, which are reflected in modified degrees of inflammation and disease severity, or from a fundamental dysregulation of inflammatory cytokine responses. To more effectively distinguish these mechanisms, we set up experiments to determine if there was a reduced inflammatory response in *Scn8a^dmu^* ^/+^ mice *in vitro* in the absence of the systemic influences of EAE or acquired immune responses. We did this by analyzing the response of primary cultured innate immune cells activated with the classical immune activator LPS. Mast cells were derived from the long term (6 to 8 week) culture of bone marrow stem cells (BMMC) and were exposed to LPS for 24 hours (35). BMMC derived from *Scn8a^dmu^* ^/+^ and *Scn8a^+/+^* mice were treated with three different concentrations of LPS (100, 10, 1 µg/mL) and the production of the classical inflammatory mediator IL-6, a major cytokine product of mast cells, was determined. We observed significantly lower levels of IL-6 in supernatants from *Scn8a^dmu^* ^/+^ BMMCs treated with 100 µg/mL LPS at 24 hours when compared with mast cells derived from wild type control littermate mice (0.2888 ± 0.0618 ng/ml, *n* = 5 vs 0.6389 ± 0.1089 ng/ml, *n* = 5, *P* = 0.044, **Figure 7**) post-treatment.

## Discussion

While the adaptive immune system has been a primary research focus in MS, the role of the innate immune system is attracting an increasing interest due to its importance for the generation of acquired immunity and for the inflammatory response that impacts the progression of the disease (36). Inflammation is a primary hallmark of MS and is characterized by the infiltration of multiple types of leukocytes into the CNS, leading to the disruption of the BBB and axonal damage (37). Immune cells secrete multiple mediators of inflammation, including cytokines, chemokines, glutamate, reactive oxygen, and nitrogen species, some of which are neurotoxic (38–40). Dendritic cell populations such as myeloid dendritic cells are well established as critical cells in the generation of EAE (41, 42). Inflammatory mediators such as TNF, IL-1 and IL-6 play a key role in regulating the mobilization of dendritic cells to draining lymph nodes. They also aid in the maturation of dendritic cells. In this way, the inflammatory response and acquired autoimmune response in EAE are intricately linked.

In MS and in the EAE model, neuronal Nav channel expression is dysregulated, and they are known to become abnormally distributed along axons following demyelination. As a result, these channels have been hypothesized to cause sodium-calcium exchangers to operate in reverse such that they extrude excess axoplasmic sodium while calcium is imported, which eventually results in neuronal death by apoptosis (18, 30). However, Nav channels, including Nav1.6 are also present in immune cells where an understanding of their role remains limited and their involvement in the etiology of MS and EAE is not well characterized. Macrophages and microglia express different types of Nav channels, and which have been implicated in different functions of these cells, including motility and migration (24, 43). In EAE and MS, Craner et al. (25) showed that the expression of Nav1.6 is upregulated in activated microglia and macrophages. Increased activation of microglia and macrophages in MS has been linked to the axonal degeneration *via* induction of phagocytosis (24, 25, 44), antigen presentation (45), cell migration (24, 26), stimulation of CD4^+^ T cell proliferation, and production of pro-inflammatory cytokines and chemokines (24, 46). To evaluate the role of Nav1.6 in the inflammatory response during EAE, we used *Scn8a^dmu/+^*, which are heterozygous for a null allele of *Scn8a* and express low levels of *Scn8a*, the gene that encodes the alpha subunit of Nav1.6. The heterozygous mice that had a single mutation in Nav1.6 resulting in a decreased expression of the channel protein did not show any overt neurological deficit; however, they presented with behavioral and emotional changes (47). It has been reported that Nav1.2 compensates for partial reductions of Nav1.6 in heterozygous mice indicating that Nav1.6 is unable to fully occupy the nodal membrane, which allows Nav1.2 to function and stabilize at the node of Ranvier. In addition, the similarity between the properties of Nav1.6 and Nav1.2 and their clustering indicates a binding competition at the node (48).

Mice were immunized with MOG_35-55_ as the antigen to induce chronic monophasic EAE (32, 49). The *Scn8a^dmu/+^* and control *Scn8a* ^+/+^ littermates were co-housed to ensure that they shared the same environment. Clinical symptoms started at day 8 post-EAE and progressed similarly in both groups, consistent with the study of Horstmann et al. (50). However, during the recovery phase (days 19-26), the clinical scores in *Scn8a^dmu/+^* mice were significantly lower than *Scn8a* ^+/+^ in which the average clinical scores were 2.5. This score indicates the ability of the mouse to walk and move around the cage relatively normally. In contrast, *Scn8a^+/+^* mice showed an average clinical score was 3, which means the mice had a significant deficit in walking and moving around the cage. *Scn8a^dmu/+^* had improved motor function during this period and, correspondingly, the weight of the *Scn8a^dmu/+^* mice was increased relative to wild type.

During EAE, peripheral blood Gr-1^+^/CD11b^+^ myeloid cells were significantly reduced in *Scn8a^dmu/+^* in the EAE chronic phase. Several studies have reported that the expansion of Gr-1^+^/CD11b^+^ cells in the peripheral blood in the early chronic phase of EAE is associated with the pathogenesis of EAE (29). Neutrophil depletion using anti-Ly6G or anti-Gr-1 antibody hinders the onset of EAE (51, 52). The infiltration of these cells into the CNS was also investigated. Gr-1^+^/CD11b^+^ cells were significantly reduced in the optic nerve of *Scn8a^dmu/+^* mice in keeping with their reduced numbers in the peripheral blood.

Dendritic cells are known to have a pivotal role in EAE (42). However, Nav 1.6 expression has not been observed on dendritic cells. In contrast it is observed on NK cells, macrophages, platelets and a number of other innate immune effector cells. A full analysis of DCs was beyond the scope of this study. A predominant, direct role for Nav1.6 on DC function would more likely affect initial phases of the disease and the development of an autoimmune response and would be less likely to selectively impact recovery and chronic phases. Many DC subsets may potentially be modified in behaviour as a result of altered IL-10 or IL-6 levels in their microenvironment.

Levels of the cytokine IL-6 were significantly reduced in the plasma of *Scn8a^dmu/+^* mice during the recovery and chronic phases of EAE. IL-6 is a hallmark cytokine of inflammation that induces the acute phase response and promotes the development of plasma cells from B cells. Notably, a higher level of CD19^+^ B cells was observed in *Scn8a^dmu/+^* mice. B cells are also implicated in the recovery phase of EAE because of their ability to produce IL-10 that resolves inflammation; however, they are not required for its initiation (53).

Ultrastructural analysis of axonal pathology in chronic phase EAE optic nerves revealed a higher g-ratio in *Scn8a^dmu/+^* mice post-EAE than *Scn8a^+/+^* mice and the distribution of the axonal area was shifted to the right in *Scn8a^+/+^* mice. In addition, based on g-ratio, we found evidence of possible remyelination occurring in *Scn8a^dmu/+^* and not in *Scn8a^+/+^* mice. In the days preceding our endpoint at 35 days post EAE, in the midst of this period of intense axonal loss, it is conceivable that more demyelinated/demyelinating axons survived in *Scn8a^dmu/+^* due to reduced Nav1.6 levels, thus providing the substrate for the observed remyelination. While in the CNS, remyelination may occur but is not sufficient to fully restore normal CNS function following demyelination (54), the partial blockade of *Scn8a* function or expression perhaps could nonetheless be sufficient to enhance function through remyelination. Determination of the specific role that Nav1.6 may have in regulating remyelination, and whether the channels involved are of neuronal or glial origin, will require further investigation.

Interestingly, the infiltration of Gr-1^+^/CD11b^+^ cells and the level of the pro-inflammatory cytokine TNF in the brain increased markedly after EAE in *Scn8a^+/+^* mice, effects that were considerably less pronounced in the *Scn8a^dmu/+^* mice. TNF has been reported to cause apoptosis of oligodendrocytes and to induce the release of glutamate from astrocytes that leads to damage of oligodendrocytes and neurons (55). It has also been reported that in cultured cortical neurons, TNF and IL-1β elevate Nav currents *via* the upregulation of TTX-sensitive Navs *via* a p38 MAPK dependent pathway (56). When combined with our results, these observations suggest the possible presence of bidirectional regulation between Nav channels and TNF. Additionally, treatment with anti-TNF-receptor IgG prevented the development of clinical signs of active EAE (57). A recent study by Ding et al. (58), has demonstrated that Nav1.6 upregulation in neuropathic pain caused by nerve injury might be through the activation of the TNF/STAT3 pathway. Our observed pathological improvements in *Scn8a^dmu/+^*, including improved motor function and reduced myeloid cell infiltration in the optic nerve, could, therefore, plausibly be a result of Nav1.6 influence on TNF expression.

In examining anti-inflammatory cytokines, we observed that the level of IL-13 was significantly increased in chronic phase EAE *Scn8a^dmu/+^* mice. Most striking, however, was the presence of dramatically higher levels of IL-10 and TGF-β1 in untreated (non-EAE) *Scn8a^dmu/+^* mice vs non-EAE *Scn8a^+/+^* controls. The Th2 subset of T cells are known to produce cytokines such as IL-4, IL-13, and IL-10 (59), which can ameliorate EAE by inhibiting Th1-like responses through the reduction of IFN-γ, TNF and IL-12 levels (60). In addition, IL-10 inhibits a variety of innate immune and inflammatory events including upregulation of adhesion molecule expression, proinflammatory cytokine and chemokine production (58). IL-13 can also promote the polarization of macrophages to less inflammatory M2 phenotype (61, 62). In our study, the strong expression of anti-inflammatory cytokines in the CNS prior to disease onset could have had a protective effect that resulted in the reduction of inflammation in chronic phase EAE *Scn8a^dmu/+^* mice.

To determine if heterozygous *Scn8a^dmu/+^* mice displayed reduced innate immune system-mediated inflammation outside of the context of EAE, the intraperitoneal LPS administration model was employed. Our results demonstrate that significantly lower neutrophil recruitment in *Scn8a^dmu^* ^/+^ than in *Scn8a^+/+^* mice at 16-hour post-LPS, suggesting that Nav1.6 may play a role in directly or indirectly facilitating migration. In this context, it is noteworthy that Craner et al. (25) has shown that the expression of Nav1.6 is upregulated in activated microglia and macrophages in response to LPS (25). Furthermore, increased activation of microglia and macrophages in MS has been linked to axonal degeneration *via* induction of pro-inflammatory cytokines (24, 25). It was shown additionally that blocking of Nav1.6 by TTX in rat microglia or the lack of Nav1.6 in microglia isolated from *Scn8a^med/med^* (Nav1.6-null) mice, both have a significant negative impact on the phagocytic function of activated microglia by 40% and 65%, respectively (25).

Mast cells are innate immune cells known to respond to LPS, their localization in the leptomeninges has suggested a possible contribution of these cells in the regulation immune cell trafficking through the BBB (63). Bone marrow-derived mast cells (BMMC) were used as an *in vitro* model of inflammatory mediator responses, which could be assessed independently from neuronal mediators and impacts. Mast cells derived from *Scn8a^dmu/+^* produced lower levels of IL-6 than cells derived from *Scn8a^+/+^* animals in response to LPS stimulation. Taken together, these results indicate that a reduction in *Scn8a* expression has a significant impact on the inflammatory response both in EAE and in response to LPS, even in isolated immune cells.

It is increasingly evident that abnormal activity and altered expression of Nav channels not only in neurons but also in immune cells are central to the pathophysiology of MS (64, 65). Our study provides evidence that reduced *Scn8a* expression *in vivo* is associated with several indicators of reduced inflammation and damage EAE including improved motor capacity during the recovery and early chronic phase, a decrease in neutrophil infiltration, reduced proinflammatory IL-6 in the blood, and decreased infiltration of myeloid cells within the optic nerve. Overall, this is the first *in vivo* study to report changes in the regulation of inflammation related to a reduced level of *Scn8a* in EAE. The decreased *in vivo* and *in vitro* response to LPS in *Scn8a^dmu/+^* we observed suggests that Nav1.6 could be a general regulator of inflammation with impacts in multiple settings and not limited to models of neuronal damage.

## Data Availability Statement

The datasets used and analyzed during the current study are available from the corresponding author on reasonable request.

## Ethics Statement

All experiments were approved by the Dalhousie University Committee on Laboratory Animals (protocol numbers 17-012 and 19-050) and carried out in compliance with the Canadian Council for Animal Care guidelines.

## Author Contributions

BA generated EAE mice, performed induction of EAE and LPS injection, tissue collection, ELISA, qPCR, flow cytometry, histological staining, data analysis, and wrote the manuscript draft. BD performed the EAE clinical score analysis, flow cytometry analysis, and helped edit early versions of the manuscript. ST and SK made the g-ratio measurements. PC assembled the figures and performed histology quantification. PC and JM designed the study and evaluated experimental results, and SK, PC, and JM critically revised draft versions of the manuscript. All authors contributed to the article and approved the submitted version.

## Funding

The Nova Scotia Research Foundation supported this work (to PC), the Dalhousie Medical Research Foundation/Gillian’s Hope MS Research Grant (to PC), The Canadian Institutes of Health Research (CIHR) grant (to JM) grant number THC-135230 and MOP-93517, and Jouf University, Saudi Arabia (to BA). BA is a recipient of a postgraduate scholarship from the Saudi Cultural Bureau (Canada) and Al Jouf University.

## Conflict of Interest

The authors declare that the research was conducted in the absence of any commercial or financial relationships that could be construed as a potential conflict of interest.
