# Supplementary figures and images for: Mice Heterozygous for the Sodium Channel Scn8a (Nav1.6) Have Reduced Inflammatory Responses During EAE and Following LPS Challenge
Source: Front Immunol. 2021 Mar 19;12:533423. doi: 10.3389/fimmu.2021.533423 (PMC8017164; doi:10.3389/fimmu.2021.533423)

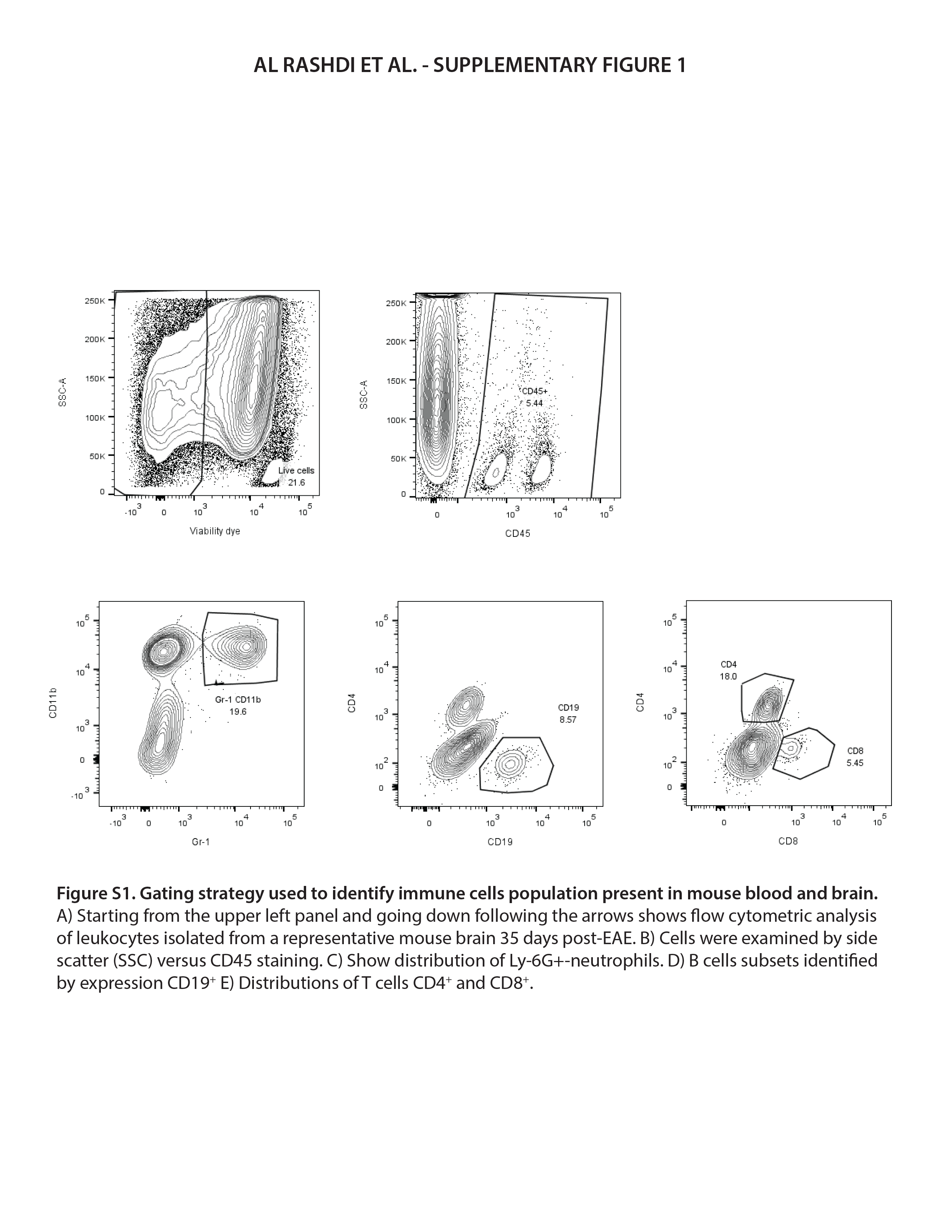

Supplement: Supplementary file 1 [file Image_1.tif]

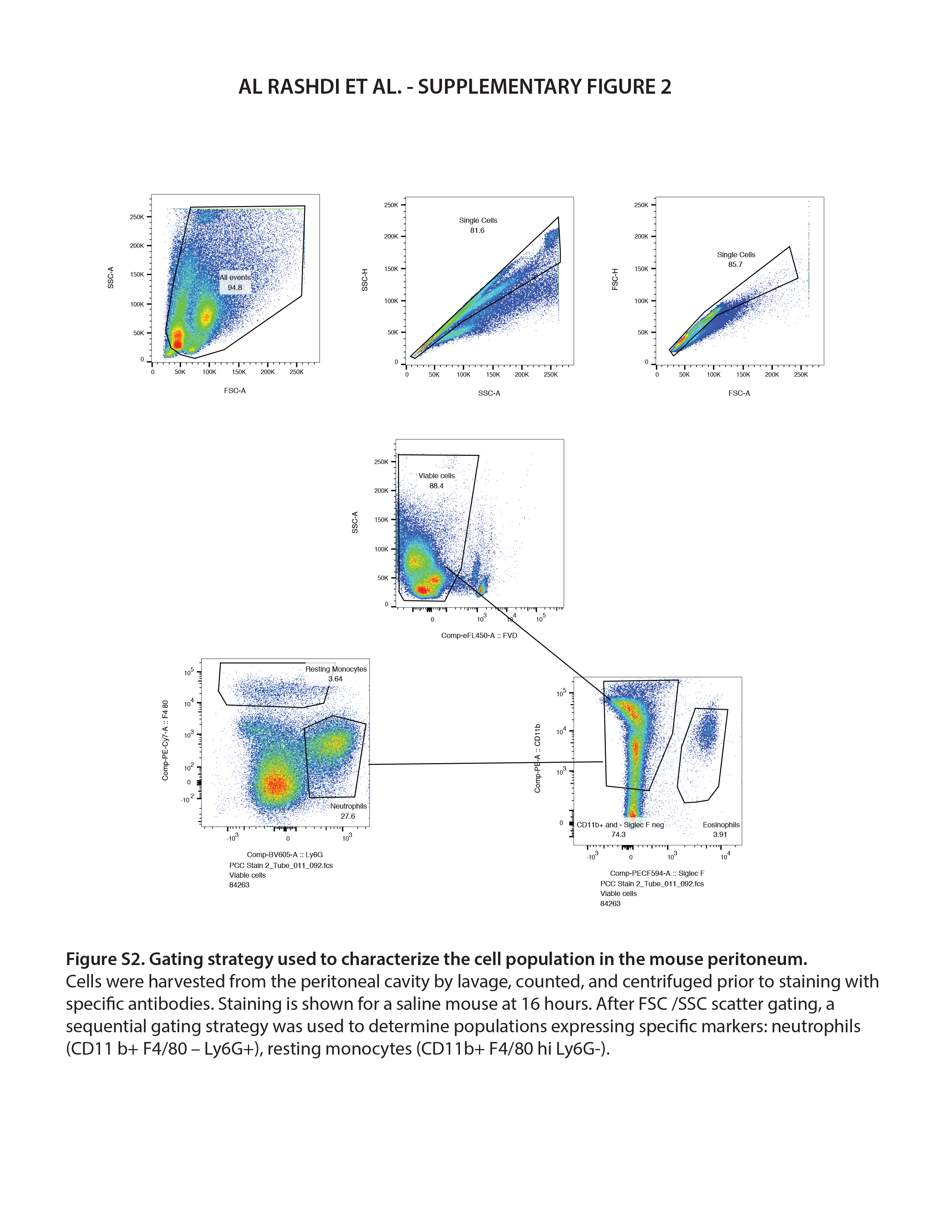

Supplement: Supplementary file 2 [file Image_2.tif]
